# Motion correction and its impact on quantification in dynamic total-body 18F-fluorodeoxyglucose PET

**DOI:** 10.1186/s40658-022-00493-9

**Published:** 2022-09-14

**Authors:** Tao Sun, Yaping Wu, Wei Wei, Fangfang Fu, Nan Meng, Hongzhao Chen, Xiaochen Li, Yan Bai, Zhenguo Wang, Jie Ding, Debin Hu, Chaojie Chen, Zhanli Hu, Dong Liang, Xin Liu, Hairong Zheng, Yongfeng Yang, Yun Zhou, Meiyun Wang

**Affiliations:** 1 Paul C. Lauterbur Research Center for Biomedical Imaging, Shenzhen Institute of Advanced Technology, Chinese Academy of Sciences, Shenzhen, People’s Republic of China; 2 Henan Provincial People’s Hospital and the People’s Hospital of Zhengzhou, University of Zhengzhou, Zhengzhou, People’s Republic of China; 3 Central Research Institute, United Imaging Healthcare Group Co., Ltd, Shanghai, People’s Republic of China; 4 School of Biomedical Engineering, Shanghai Tech University, Shanghai, People’s Republic of China; 5 United Imaging Research Institute of Innovative Medical Equipment, Shenzhen, People’s Republic of China

**Keywords:** Motion correction, Total-body PET, Dynamic imaging, Kinetic modeling

## Abstract

**Background:**

The total-body positron emission tomography (PET) scanner provides an unprecedented opportunity to scan the whole body simultaneously, thanks to its long axial field of view and ultrahigh temporal resolution. To fully utilize this potential in clinical settings, a dynamic scan would be necessary to obtain the desired kinetic information from scan data. However, in a long dynamic acquisition, patient movement can degrade image quality and quantification accuracy.

**Methods:**

In this work, we demonstrated a motion correction framework and its importance in dynamic total-body FDG PET imaging. Dynamic FDG scans from 12 subjects acquired on a uEXPLORER PET/CT were included. In these subjects, 7 are healthy subjects and 5 are those with tumors in the thorax and abdomen. All scans were contaminated by motion to some degree, and for each the list-mode data were reconstructed into 1-min frames. The dynamic frames were aligned to a reference position by sequentially registering each frame to its previous neighboring frame. We parametrized the motion fields in-between frames as diffeomorphism, which can map the shape change of the object smoothly and continuously in time and space. Diffeomorphic representations of motion fields were derived by registering neighboring frames using large deformation diffeomorphic metric matching. When all pairwise registrations were completed, the motion field at each frame was obtained by concatenating the successive motion fields and transforming that frame into the reference position. The proposed correction method was labeled SyN-seq. The method that was performed similarly, but aligned each frame to a designated middle frame, was labeled as SyN-mid. Instead of SyN, the method that performed the sequential affine registration was labeled as Aff-seq. The original uncorrected images were labeled as NMC. Qualitative and quantitative analyses were performed to compare the performance of the proposed method with that of other correction methods and uncorrected images.

**Results:**

The results indicated that visual improvement was achieved after correction of the SUV images for the motion present period, especially in the brain and abdomen. For subjects with tumors, the average improvement in tumor SUVmean was 5.35 ± 4.92% (*P* = 0.047), with a maximum improvement of 12.89%. An overall quality improvement in quantitative K_i_ images was also observed after correction; however, such improvement was less obvious in K_1_ images. Sampled time–activity curves in the cerebral and kidney cortex were less affected by the motion after applying the proposed correction. Mutual information and dice coefficient relative to the reference also demonstrated that SyN-seq improved the alignment between frames over non-corrected images (*P* = 0.003 and *P* = 0.011). Moreover, the proposed correction successfully reduced the inter-subject variability in K_i_ quantifications (11.8% lower in sampled organs). Subjective assessment by experienced radiologists demonstrated consistent results for both SUV images and K_i_ images.

**Conclusion:**

To conclude, motion correction is important for image quality in dynamic total-body PET imaging. We demonstrated a correction framework that can effectively reduce the effect of random body movements on dynamic images and their associated quantification. The proposed correction framework can potentially benefit applications that require total-body assessment, such as imaging the brain-gut axis and systemic diseases.

## Introduction

Patient motion is a long-standing problem in positron emission tomography (PET) imaging. Two types of movements, namely physiological movements (internal organ deformation, e.g., heart beating and respiration) and non-physiological movements (random changes in the body or head position), contribute to PET image degradation [1]. Motion will be even more problematic when an accurate quantification is required such as for assessing treatment using absolute change in SUV and other kinetic parameters [2–4]. Motion is even more problematic for the high-resolution scanners under development as they are vulnerable to subtle movements. As the first commercial total-body scanner, uEXPLORER has the capability of imaging the whole body simultaneously [5]. It has ultra-high sensitivity compared with conventional scanners and a resolution of 2.9 mm in the center of the axial direction [6]. Dynamic imaging is essential for utilizing the full potential of such systems but could be vulnerable to patient movement, e.g., a patient may have trouble staying still for 60 min or more, which in turn affects not only the visual quality but also the accuracy of the quantification across the entire body [7, 8].

Attempts have been made to reduce the scan time in total-body PET imaging, which in turn reduces the likelihood of motion [9, 10]. Another way to reduce the motion is patient immobilization and coaching; this involves communicating the importance of remaining still to the patient or providing training with devices for immobilization. However, a downside of these prospective methods is that they often fail to eliminate the motion. Therefore, investigating motion correction approaches is important [11].

As stated earlier, physiological and non-physiological body motion are the two main types of patient motion. Researchers have previously studied extensively how to correct cardiac and respiratory motion in the thorax [12–17]. On the other hand, although body motion can be minimized by patient cooperation, it remains a challenging problem in PET. Most studies to resolve body motion focused on brain or cardiac PET static imaging [18–22]. Other researchers attempted to resolve abdominal movement on PET imaging by using MRI information simultaneously acquired in a PET / MRI session [23–26]. On the contrary, few studies have attempted to compensate for the effect of whole-body movement in PET imaging, especially for dynamic total-body imaging. One reason of course is that a conventional scanner with a multi-bed protocol cannot truly simultaneously acquire the whole-body data. Motion tracking is an attractive option [27] that has been proven effective for monitoring brain and respiratory movement but may struggle in tracking whole-body motion due to the complex nature of the movement. An integrated PET/MR solution is also not feasible as a total-body PET/MR scanner is required but does not exist yet.

In this study, we proposed an image-based correction framework that can compensate for body movement in dynamic total-body PET imaging. We focused on random body shifts and deformation across the entire body, but not periodic respiratory or cardiac movements. Little movement is assumed at the early stage of a scan for the subjects included in this study, and the data from the subsequent period can be aligned to the reference position. In the following context, we will first illustrate the implementation and then demonstrate the evaluation. Discussion about the results, limitations and future work will follow.

## Materials and methods

### Patient population and data acquisition

We studied 12 dynamic 18F-fluorodeoxyglucose (FDG) scans acquired on a uEXPLORER PET/CT (United Imaging Healthcare, Shanghai, China) from December 2020 to July 2021 at Henan Provincial People’s Hospital. Detailed information on each subject is presented in Table 1. The exact location of the tumor lesion (if present) is also reported. The study was approved by the local Ethics Committee, and written consent was obtained from each subject before the scan. The scanning workflow and data formatting are as follows. A CT scan was first performed for attenuation correction. Then, a 60-min long list-mode acquisition was initiated with the bolus intravenous injection of 18F-FDG at the ankle. List-mode data were binned into 66 frames (5 s × 24, 10 s × 6, 30 s × 6, 60 s × 6 and 120 s × 24) and reconstructed on the scanner workstation into a 192 × 192 × 673 matrix with a voxel size of 3.125 × 3.125 × 2.866 mm^3^ by using the 3D ordered subset expectation–maximization algorithm (with TOF and PSF, 3 iterations, 28 subsets, 2-mm Gaussian post-smoothing). Attenuation and scatter corrections were performed during the reconstruction with the CT-based attenuation maps.

**Table 1:** Patient demographics

Subject (lesion)	Gender	Age	Height (cm)	Weight (kg)	Injection dose (MBq)
001 (N/A)	M	27	166	60	220
002 (lung)	F	57	166	61	242
003 (lung)	M	28	174	75	302
004 (N/A)	M	31	170	72	289
005 (liver)	M	47	178	105	387
006 (N/A)	F	35	164	63	233
007 (N/A)	F	30	162	73	294
008 (gastrointestinal)	M	24	182	99	366
009 (N/A)	M	57	175	73	278
010 (lung)	F	49	165	75	301
011 (N/A)	M	55	173	80	218
012 (N/A)	F	58	168	60	261
Mean ± s.d	7 M, 5F	41.5 ± 12.9	170.2 ± 5.9	74.6 ± 14.4	282.5 ± 53.4

Inter-frame positional changes were visually identified for all scans. The motion mostly happened after 10 min in the scans included in this study. For each scan, 10–60-min list-mode data were again reconstructed into fifty 1-min-long frames by using the same reconstruction parameters as above. This will allow a better temporal sampling in reconstructed frames to facilitate the motion correction. As a result, a total of 90 frames (5 s × 24, 10 s × 6, 30 s × 6 and 60 s × 54) constituted the new set of dynamic images. From this new set of images, the image-derived input function (IDIF) was extracted from the ascending aorta by drawing a 10-mm-diameter ROI on six consecutive slices in an image obtained by combining early time frames (0–60 s), where the effects of motion and partial volume were less prominent than in the left ventricle [28]. The uptake difference in blood and plasma was not accounted for in this study.

### Implementation of the proposed correction framework

The registration of dynamic frames was performed sequentially in a manner similar to what has been proposed for dynamic brain PET [29, 30]. We assumed there is little movement in the early stage (0–10 min), hence the associated frames remained unchanged. The last frame within the first 10 min (frame 40) was used as the reference image. Multiple pairwise registrations were initiated from frame 41 onwards, and the subsequent frames were aligned to the reference position by matching each to its previous frame. In this manner, the potential contrast difference between frames is less problematic for pairwise registration as in conventional image-based methods. Thus, similarity metrics such as normalized cross-correlation can be employed.

The pairwise registration between neighboring frames was performed as follows. For total-body PET imaging, the human body may shift and deform randomly in time and space, resulting in non-uniform intensity change across the whole body. This poses challenges in aligning two neighboring frames when compared with applications such as brain PET. Therefore, we employed large deformation diffeomorphic metric matching (LDDMM), which is designed for large space–time deformation. LDDMM is a non-parametric approach based on principles from fluid mechanics [31, 32] and has been successfully applied to many medical imaging applications [33–35]. Here the goal is to register the source image *I* and target image *J* (neighbor of *I*) by finding a time-varying field $v$ that minimizes the energy function:

1 $$E(v) = M(I,J,\phi_{1,0} ) + w\int_{0}^{1} {\left\| {Lv_{t} } \right\|}_{2} dt$$

where $M(I,J)$ is the similarity measure, which is normalized cross-correlation. The second L2-norm term penalizes the non-smooth velocity fields, and *L* is the differential operator. $\phi$ parameterizes a family of diffeomorphisms that can be generated by integrating a smooth velocity field, $v:\Omega \times t \to {\mathbb{R}}^{d}$, using an ordinary differential equation:

2 $$\frac{d\phi (x,t)}{{dt}} = v(\phi (x,t),t),\,\,\,\,\phi (x,0) = x$$

Exploiting the fact that diffeomorphism $\phi$ can be decomposed into two components $\phi$_1_ and $\phi$_2,_ we constructed a symmetric alternative to Eq. (1); accordingly, the optimization problem can be expressed as

3 $$\{ v_{1}^{*} ,v_{2}^{*} \} = \mathop {\arg \min }\limits_{{v_{1} ,v_{2} }} \left\{ {\int_{\Omega } M \left( {I \circ \phi_{1} (x,0.5),J \circ \phi_{2} (x,0.5)} \right)d\Omega + w\left( {\int_{0}^{0.5} {\left\| {Lv_{1} (x,t)} \right\|^{2} dt + \int_{0.5}^{1} {\left\| {Lv_{2} (x,t)} \right\|^{2} } dt} } \right)} \right\}$$

The goal is to find $v_{1}$ that minimizes the variational energy from *t* = 0 to 0.5, whereas $v_{2}$ minimizes from *t* = 1 to 0.5. Gradient-based iterative updates deform $I$ and $J$ along the geodesic diffeomorphisms $\phi$ to a midway fixed point, thus motivating the denotation of the registration strategy as symmetric normalization–SyN [32]. A CPU implementation of SyN is available in the ANTs package. Given a large number of voxels in a total-body PET image, we implemented it on a GPU (Nvidia GTX 2080Ti).

Upon finishing all pairwise registrations, the motion field at each frame was obtained by concatenating successive motion fields from all previous frames. The motion-compensated frame was thus obtained by transforming the image with the derived motion fields. For a given scan, the above process will eventually generate a new set of frames aligned to the reference position defined at the early scan. We labeled this correction framework as SyN-seq in the following context. After correction, the 90-frame of dynamic images were summed back to 66 before performing subsequent analysis.

### Comparison of different methods

The original uncorrected images were labeled as NMC. The method that performed the diffeomorphism registrations to align each frame to a designated reference frame was labeled as SyN-mid, where the reference was selected as the mid-frame (frame 70). The registration parameters were similar to the ones used for SyN-seq, except that the similarity metric was normalized mutual information to account for the potential large contrast difference among the dynamic images.

Instead of SyN, the method performed the sequential affine registration was labeled as Aff-seq. Affine registration was used to model and estimate the motion as global transformation (represented as translations, rotations and scaling). The optimization in the transformation parameters started from a coarse scale, which was then used to initialize the registration at the next finer scale. Similar to SyN-seq, the similarity metric was selected as normalized cross-correlation to account for the image dissimilarity. Powell algorithm [36] was used for optimization with a fractional tolerance of 10^−4^ and 500 maximum running iterations. The coarse-to-fine registration was repeated (three levels in total) until the finest scale and stopping criteria were reached.

### Data analysis

**Fig. 1:**
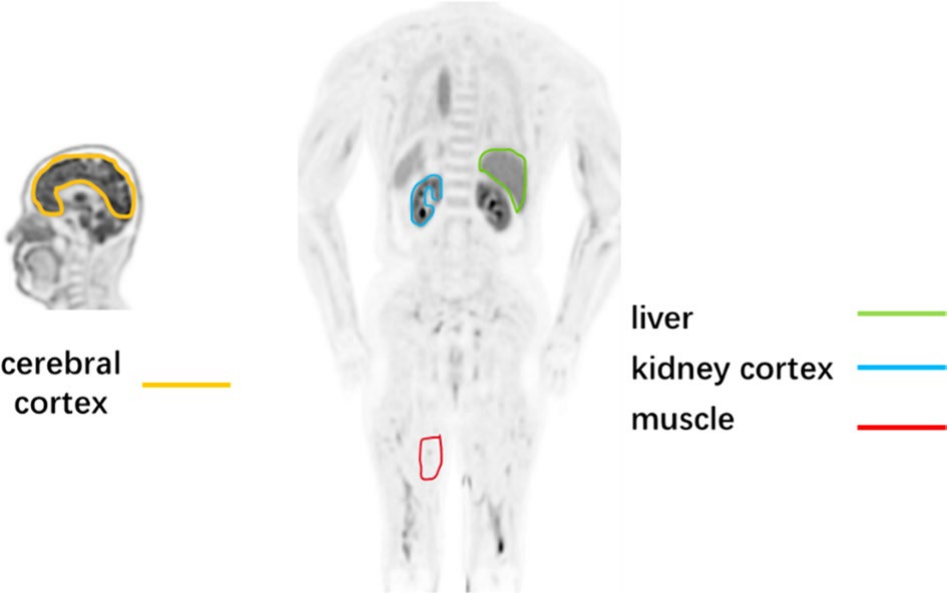
Example ROIs to extract the time-activity curves that are used for quantifications

All statistical analyses were performed using the Statistical and Machine Learning Toolbox in MATLAB R2018b. The common arbitrary threshold of 0.05 was selected as the level of significance. The proposed correction framework SyN-seq was compared with SyN-mid, Aff-seq and NMC. For all 12 subjects, visual and quantification assessments were performed as described below. Motion-contaminated 10-min SUV images were created by combining the corresponding frames. For the five patients with solid tumors, the lesions were delineated using a semiautomatic region growing method with a 50% cutoff threshold of the maximum intensity value, and their SUVmean were reported before and after correction. Mutual information and dice coefficient were computed between each transformed frame and the reference to reveal the degree of recovery. Sampled time–activity curves (TACs) of the cerebral cortex, kidney cortex, liver and thigh muscles (ROIs in Fig. 1) were plotted and compared. Parametric K_i_ and K_1_ images were computed for each set of images by using fast nonlinear estimation under the assumption of an irreversible two-tissue compartment model [37]. The IDIFs were measured for each set separately, as described earlier in the Methods section. The fitting residual (FR) was computed as an indication of the goodness of fit, which should be low for the dynamic images to be better aligned:

4 $$FR = \sum\limits_{k \in F} {\sum\limits_{j \in N} {\left( {I_{k} (x_{j} ) - \overset{\lower0.5em\hbox{$\smash{\scriptscriptstyle\frown}$}}{I}_{k} (x_{j} ,p)} \right)}^{2} }$$

where *k* and *F* are the index and total number of frames, respectively; *j* and N are the index and total number of voxels in a frame, respectively; and *p* denotes the fitted parameters.

To determine the inter-group variety, coefficient of variation (CV) in K_i_ and K_1_ was computed for the cerebral cortex, kidney, liver and thigh muscle; the low value indicates the low variation in the group:

5 $${\text{CV }} = {\text{ SD}}/{\text{mean}}$$

where mean and SD were computed for all subjects. A small CV value indicates low inter-subject variability in the group, thus indicating a better recovery of kinetic parameters from the effect of motion on the difference in patients.

Finally, two experienced radiologists conducted five-scale scoring for the quality of SUV and dynamic frames. The raters were blinded to the clinical information of the patients. For an SUV image, the score was determined according to the amount of artifacts and image resolution. As such, a successful motion correction should remove the positioning change when displaying dynamic frames as a movie, and a higher score will be given.

## Results

**Fig. 2:**
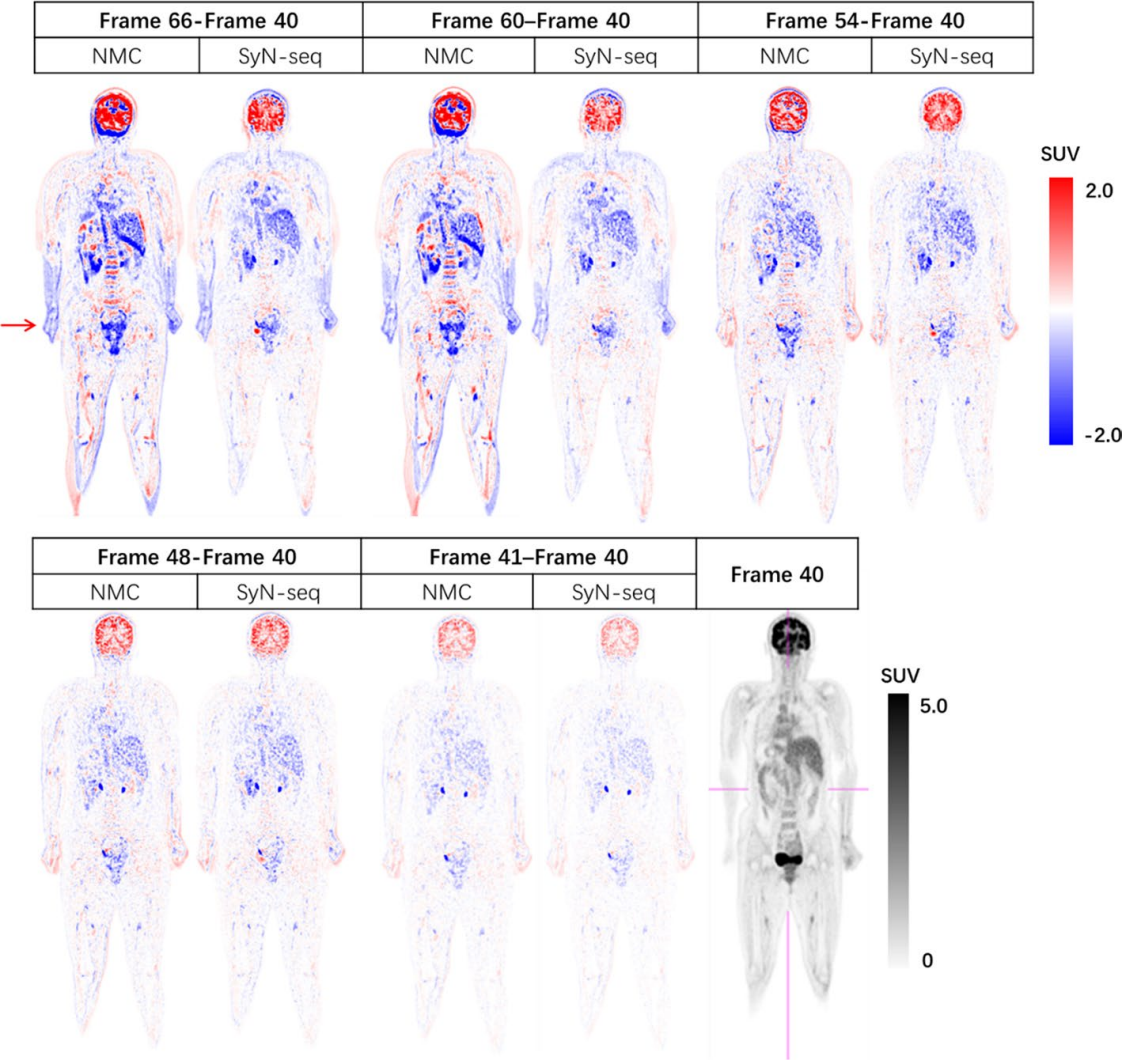
Patient 12: subtraction images at given frames before (NMC) and after correction with the SyN-seq. The corrected frames have less positional difference to the reference frame 40

**Fig. 3:**
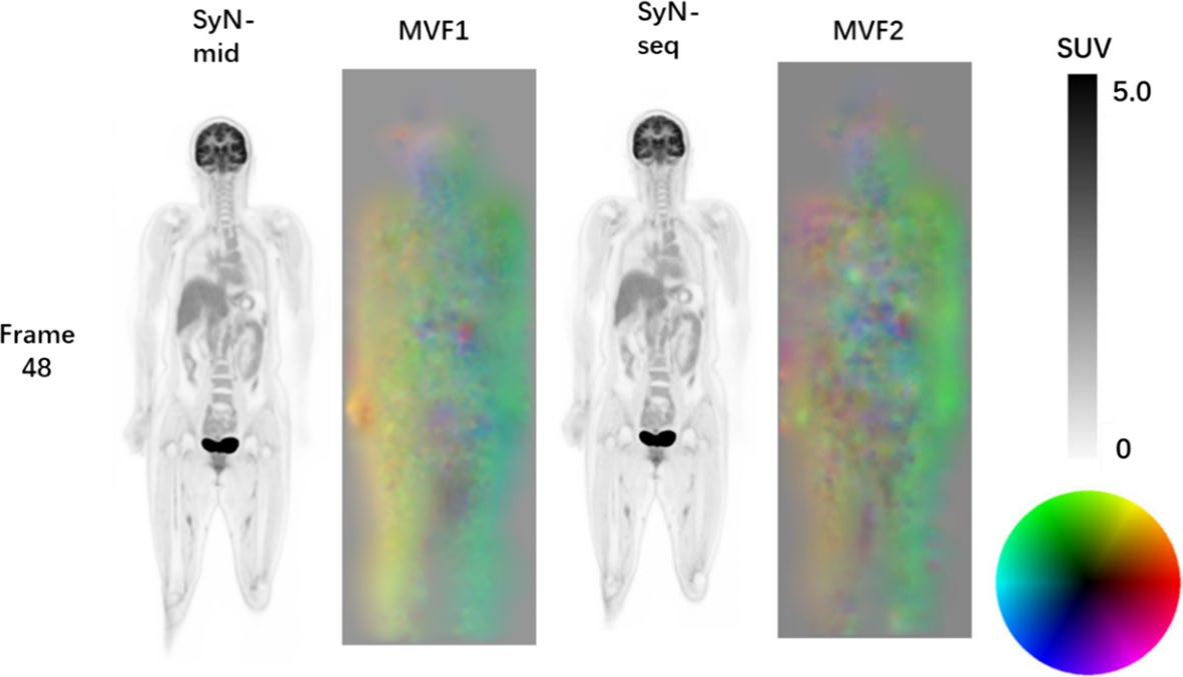
Patient 12: SyN-mid- and SyN-seq-corrected images and their associated motion fields (at frame 48). For the motion vector fields, the red, green and blue represents the three directions of the vector, and the saturation represents magnitude of the vector along each direction (as the color wheel shows)

SyN-seq required an average processing time of 35 min to process a dynamic scan. In contrast, SyN-mid and Aff-seq required average of 60 min and 8 min, respectively. The selected subtraction images (to reference frame 40) at frame 66, 60, 54, 48, 41 are shown in Fig. 2. All frames after alignment demonstrated less intensity difference than the uncorrected ones. The residual contrast difference is probably mostly due to the tracer kinetics as the distribution has not reached equilibrium yet (indicated by the subtraction image between frame 41 and 40). Note that the correction failed to compensate for the effect of arm movement due to the truncation in the transaxial plane (arrow). (Fig. 3).

**Fig. 4:**
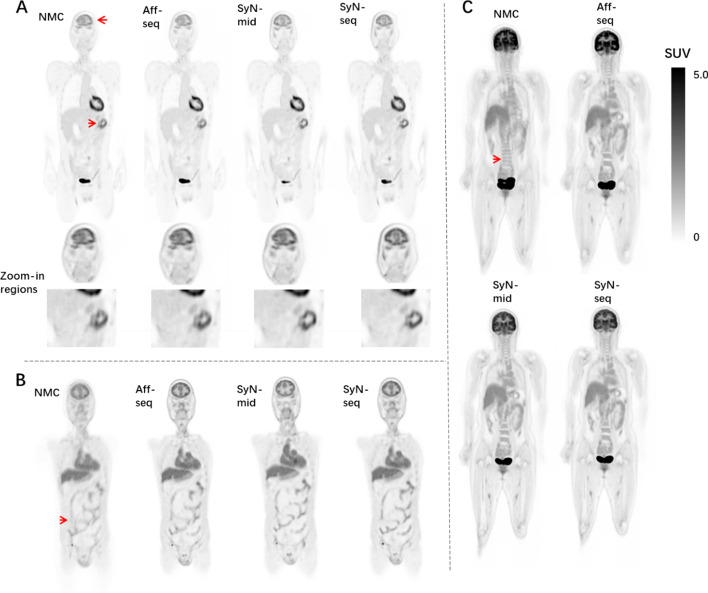
Effect of motion correction on SUV images for **A** patient 8 (50–60 min), **B** patient 9 (7–12 min) and **C** patient 12 (40–50 min). Red arrows indicate the motion-contaminated regions. The images in **A** and **B** suffered more from head and internal abdominal movement, while the one in **C** suffered from the irregular body shift mostly along the Superior-Inferior direction

In Fig. 4, SUV images from NMC, Aff-seq, SyN-seq and SyN-mid are displayed in parallel for comparison. For most motion-contaminated scans, Aff-seq recovered the image quality to some extent, while SyN-seq and SyN-mid further removed the residual artifacts. Furthermore, visual appearance of SyN-seq and SyN-mid was comparable, although there are some visual differences in abdominal and other regions. This may partly be explained by the difference in motion fields (Fig. 3) that result in the positional difference for viewing. The average improvement in tumor SUVmean was 5.35 ± 4.92% (*P* = 0.047) with a maximum improvement of 12.89%. Details effects of correction on tumor uptake are listed in Table 2.

**Table 2:** Effects of correction on tumor SUVmean for the subject with lesions

Subject (time)	NMC	Aff-seq	SyN-seq	improve percentage
002 (40–50 min)	9.58	9.64	**9.80**	2.26%
003 (50–60 min)	4.82	4.94	**5.23**	5.86%
005 (40–50 min)	**13.51**	13.41	13.49	-0.13%
007 (20–25 min)	6.05	5.98	**6.83**	12.89%
008 (50–60 min)	10.92	11.13	**11.56**	5.86%
Mean ± s.d	8.98 ± 3.55	9.02 ± 3.52	9.38 ± 3.37	5.35 ± 4.92%

The number in bold is the largest one among the group

**Fig. 5:**
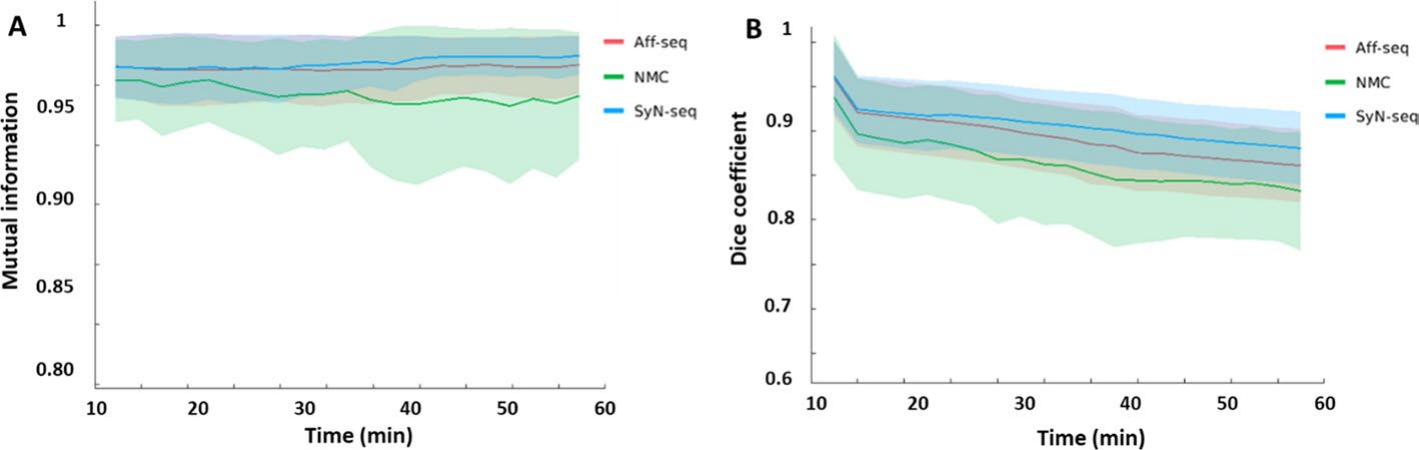
Distribution plots of the mutual information **A** and dice coefficient **B** for assessing the frame alignment. The lines represent the means, and the shadowed areas represents the associated standard deviations

**Fig. 6:**
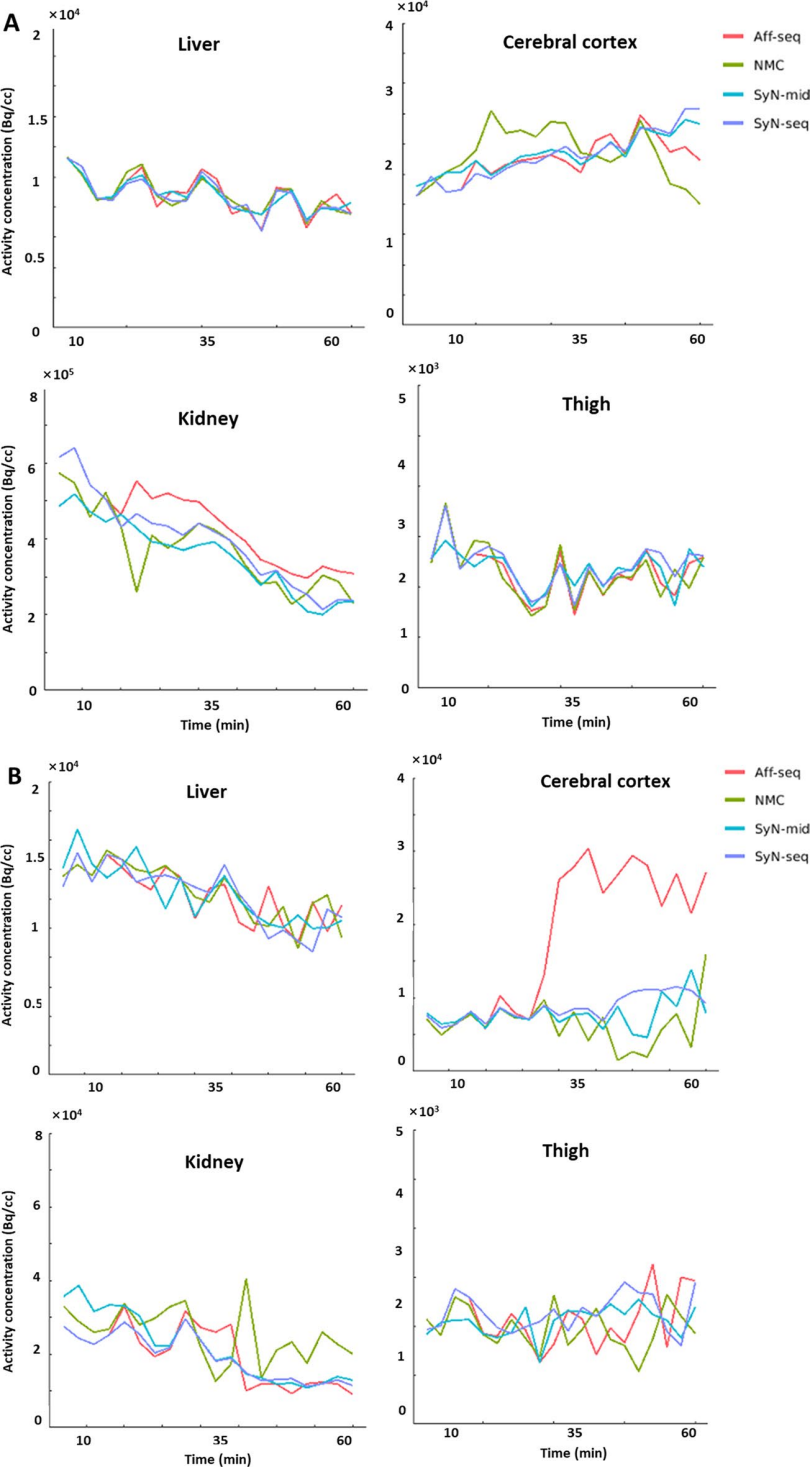
TACs sampled at organs from **A** patient 4 **B** patient 12. The quantification in the cerebral cortex and kidney cortex is more sensitive to movement, while the relatively uniform regions in liver and thigh muscle are affected to a less extent

**Fig. 7:**
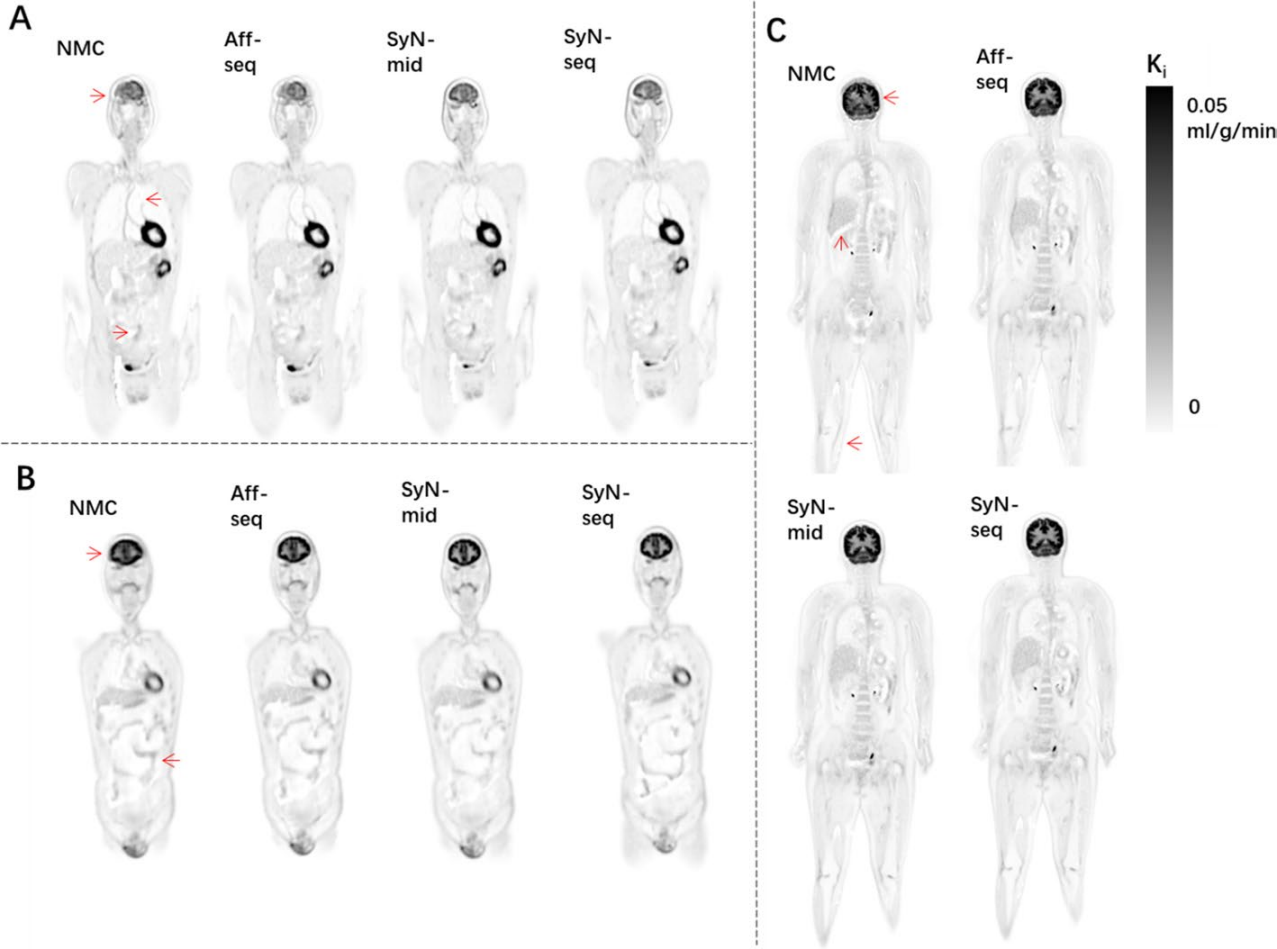
Corresponding K_i_ images for **A** patient 8, **B** patient 9 and **C** patient 12 in Fig. 4. Red arrows indicate the motion-contaminated regions, which can be different from the ones in Fig. 4

**Fig. 8:**
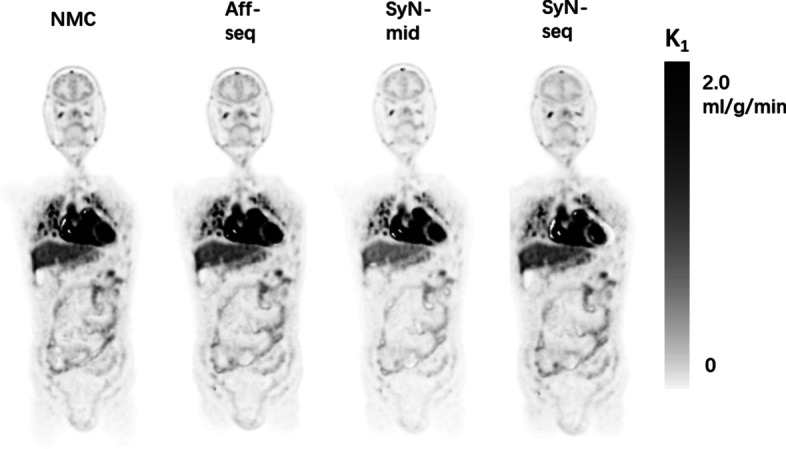
K_1_ images did not show visual differences before and after correction (corresponding to Fig. 7B). K_1_ images from other subjects have similar conclusions

SyN-seq and SyN-mid achieved the best recovery in aligning dynamic frames. Mutual information and dice coefficient at all frames relative to the reference are shown in Fig. 5. The similarity between the frames improved greatly after correction. Differences in mutual information and dice coefficient before and after correction using SyN-seq were significant (*P* = 0.003 and *P* = 0.011). There was also a significant difference between SyN-seq and Aff-seq (*P* = 0.012). However, there was no significant quantitative difference between SyN-seq and SyN-mid. Sampled TACs from patient 4 and patient 12 are displayed in Fig. 6. The quantifications in the cerebral cortex and kidney cortex were affected by the patient’s movement in late frames, whereas those in the liver and muscle were affected to a lower extent as spillover was less prominent in the uniform region. The clear anomaly from the Aff-seq profile in Fig. 6B is due to the failure of the registration at frame 40, which causes all subsequent registrations to a wrong reference position. Parametric K_i_ images are shown in Fig. 7. Similar to SUV images in Fig. 4,  Aff-seq and SyN-mid recovered the image quality to some extent, while SyN-seq further improved the image quality. The residual fitting errors when performing the kinetic modeling were 3.53 ± 0.46, 3.34 ± 0.41, 2.74 ± 0.49 and 2.88 ± 0.42 (× 10^6^ Bq/ml) for NMC, Aff-seq, SyN-seq and SyN-mid, respectively. Quantification in K_1_ exhibited little improvement after correction as shown in Fig. 8 because K_1_ mostly depends on the early phase of the scan for which no correction was applied.

Table 3 shows that both SyN-seq and SyN-mid reduced the group CV of K_i_ in the cerebral cortex, liver, kidney cortex and thigh muscle (with a mean reduction of 28.9%, 5.3%, 11.5%, 1.8%, respectively), thus demonstrating the correction successfully reduced the inter-subject variability. This reduction was not much different between SyN-seq and SyN-mid. The average score assessed by two radiologists on the SUV images were 3.5, 3.6, 4.05 and 4.0 for NMC, Aff-seq, SyN-seq and SyN-mid, respectively. The average scores on the corresponding dynamic frames were 3.2, 3.6, 4.25 and 4.23, respectively. The subjective assessment was consistent with the results in previous paragraphs.

**Table 3:** Summary of coefficient of variation (CV) and associated variances for kinetic parameters in the cerebral cortex, liver, kidney cortex and thigh muscle for 12 subjects

		NMC	Aff-seq	SyN-seq	SyN-mid
K_i_	cerebral	0.511 (0.0214)	0.395 (0.0189)	0.363 (0.0169)	**0.362** (0.0172)
	liver	0.486 (0.0023)	0.513 (0.0025)	**0.460** (0.0022)	0.469 (0.0023)
	kidney	0.522 (0.0187)	0.590 (0.01203)	**0.480** (0.0113)	0.550 (0.0114)
	muscle	0.221 (0.0004)	0.230 (0.0004)	**0.217** (0.0004)	0.220 (0.0004)
K_1_	cerebral	**0.349** (0.1739)	0.350 (0.1789)	0.350 (0.1760)	0.352 (0.1782)
	liver	0.538 (0.0081)	**0.492** (0.007)	0.500 (0.0076)	0.500 (0.0088)
	kidney	0.209 (0.7026)	0.221 (0.7221)	0.241 (0.6259)	**0.198** (0.6089)
	muscle	0.285 (0.0055)	**0.275** (0.0052)	0.283 (0.0053)	0.286 (0.0059)

The number in bold is the smallest among the group

## Discussion

In this paper, we proposed a framework to compensate for the body shift and deformation in dynamic total-body PET imaging. The proposed method is an image-based approach that attempts to align the densely reconstructed frames (1 min per frame). The results showed that the quality of SUV and K_i_ images improved after correction, and the degree of recovery varied across the subject. The tumor uptakes were also improved after correction across the subjects with tumors. The performance of SyN-seq was better than that of other correction methods in terms of quantification, although SyN-seq only outperformed SyN-mid slightly in some subjects. The results demonstrate that not only the single-subject staging accuracy can potentially be improved, and the inter-subject group variability can be reduced which in turn reduces the number of subjects required in a cross-sectional study. The successful motion correction in dynamic total-body imaging will benefit applications that require simultaneous multi-organ imaging, such as imaging of the brain-gut axis [1, 38]. When computing K_i_, we applied nonlinear estimation based on the irreversible two-tissue compartment model. Similar results are expected for the K_i_ image estimated using graphical Patlak analysis that will also benefit from motion correction. Note that the visual comparison between SyN-mid and others may not be entirely fair, as they are aligning to a different position; hence, their views in Fig. 4 and Fig. 7 may slightly be different.

We focused on random body shifts and deformation across the entire body, but not periodic physiological movements, e.g., respiratory motion. The difference between respiratory motion correction and total-body dynamic motion correction is three-fold. Firstly, most respiration is periodic; hence, there are a limited number of motion fields, which can be estimated from a static scan by existing vendor software. While a total-body dynamic scan time is relatively longer, motion correction needs to take care of the multiple random estimation of motion fields. Secondly, short-time frame registration can be more challenging than respiratory motion correction with more counts. In addition, the large contrast difference between the early and late frames poses challenges. Thirdly, we think that respiratory motion fields can be represented with a limited number of parameters, which can be well estimated by B-spline or optical flow-based algorithms [16, 39, 40]. On the other hand, whole-body motion fields are arguably more complex and require a more complex representation such as diffeomorphism. In fact, we tried B-spline registration to replace SyN in our application, but it performed worse. Despite of above difference, it would be of great interest to correct both physiological respiration and non-physiological body motion simultaneously, which may be enabled by uEXPLORER as the frames can be reconstructed at a very high temporal resolution (down to 100 ms) [5] to capture the motion in a short period.

In this study, we did not compensate for the motion at the early stage (first 10 min) because patients cooperate and stay still at the beginning but tend to move at later stages in the included scans. If the patient does move during the first 10 min, compensating for the motion becomes necessary, and we expect to see the change in K_1_ quantification after correction [41]. In such cases, SyN-mid will likely fail for some very-early frames due to the dissimilarity between them and the reference image, considering the difficulty in selecting an optimal reference frame. For a similar reason, a non-FDG tracer with fast kinetics can also challenge the effectiveness of SyN-mid. On the other hand, SyN-seq might fail in the case where the accumulation of registration errors in the sequential registration process. Another source of error could happen when registration failed for a large body movement happens in the middle of a scan, or certain frames are severely contaminated by the intra-frame motion [42]. As a consequence, the associated error will also propagate into the next registrations.

The proposed SyN-seq required an average processing time of approximately 35 min for a dynamic total-body scan on a GPU. The exact time varied for each scan, depending on the difficulty in registrations. Applying a neural network-based implementation of SyN to further reduce the computation time [43] was shown equally effective in brain MRI registrations compared with the regular diffeomorphism-based methods. However, the aim here is to demonstrate the possibility and the necessity of dynamic total-body motion correction. Therefore, the validated original SyN registration method was applied. Related to the above discussion, a recent paper proposed an unsupervised automatic deep learning-based framework to correct inter-frame body motion [44]. The motion estimation network utilizes dynamic temporal features and spatial information to produce the image registration tasks. However, one common problem, at least for a supervised method, is that it often requires a large number of training datasets for each target tracer under similar scan configurations.

Despite the encouraging results, this work is not free of limitations. Firstly, we did not consider the attenuation correction effect. It is known that motion can cause a mismatch between the attenuation map and emission data; thus, attenuation correction can induce quantification error in the reconstructed image [45, 46]. However, the goal here was to propose an automatic image-driven method that can readily work on the dynamic reconstructed images. In the future, we will investigate the possibility of accurate attenuation correction to the existing workflow once the vendor supports reconstruction with a user-defined AC map. Secondly, the potential degradation from the intra-frame motion was ignored. Although most motion artifacts were successfully suppressed, residual artifacts could still be present in a 1-min frame image. To deal with this, motion detection is required to first detect the exact time when the body starts to move and then separate the raw data in time accordingly. This will also reduce the required number of the image registration process. Thirdly, as stated earlier, SyN-seq might be inaccurate in the case when registration errors propagated in the sequential registration processes. Although we did not observe such effect in this study, caution should be taken especially when one attempts to register low-quality frames. Lastly, we expect the method will also benefit for first 10-min scan at least better than conventional image-based method, but this remains to be investigated. Similarly, the varying contrast can be problematic for a non-FDG tracer. The kinetics and the distribution of a non-FDG tracer could be quite different from FDG, which may prohibit accurate whole-body registration due to insufficient common information in-between frames. Therefore, further validations of the proposed correction on non-FDG tracers are warranted.

## Conclusion

In this work, we demonstrated an image-based motion correction method for dynamic total-body PET imaging. The proposed method can reduce the effect of body shift and deformation on image quality and quantification of both static and parametric images. The quality improvement in the total-body PET will benefit clinical applications that require simultaneous imaging at whole-body level. We plan to evaluate the feasibility of applying the proposed correction framework to certain applications, e.g., assessing the whole-body tumor burden with dynamic imaging.

## Data Availability

The data and material will be available upon reasonable request.

## Abbreviations

SUV: Standardized uptake value
FDG: Fluorodeoxyglucose
CT: Computerized tomography
3-D: Three-dimensional
4-D: Four-dimensional
OSEM: Ordered subset expectation–maximization
IDIF: Image-derived input function
HCT: Hematocrit
LDDMM: Large deformation diffeomorphic metric matching
SyN: Symmetric normalization
TAC: Time–activity curve
NMC: Non-motion corrected
CV: Coefficient of variance
CPU: Central processing unit
GPU: Graphics processing unit
